# Quantifying groundwater storage dynamics during the 2024 flood season using GRACE-FO and multi-source hydrological data in Hunan Province, China

**DOI:** 10.1038/s41598-026-48703-z

**Published:** 2026-05-17

**Authors:** Fan Lei, Jingwen Zhou, Kaijun Yang, Jide Wei, Li Cao, Fang Hu, Zhe Zhang, Yulong Zhong

**Affiliations:** 1 The Second Surveying and Mapping Institute of Hunan Province, Changsha, 410029 China; 2 Key Laboratory of Natural Resources Monitoring and Supervision in Southern Hilly Region, Ministry of Natural Resources, Changsha, 410029 China; 3 School of Geography and Information Engineering, China University of Geosciences (Wuhan), Wuhan, 430078 China

**Keywords:** GRACE-FO, Groundwater storage anomaly, Reservoirs and Dongting Lake, Flood season, Hunan Province, Environmental sciences, Hydrology, Water resources

## Abstract

Groundwater is a vital component of the hydrological cycle, and understanding its dynamics is crucial for water resource management under climate change. This study employs GRACE-FO satellite data to assess groundwater storage (GWS) dynamics in Hunan Province during the 2024 flood season (April-September). Given the abundant surface water resources in this region, we explicitly incorporate the water storage of Dongting Lake and 28 large reservoirs when calculating surface water storage anomaly (SWSA), which is crucial for estimating the GWS anomaly (GWSA). Accordingly, GWSA is obtained by subtracting the soil moisture storage anomaly (SMSA) and SWSA from the GRACE-FO-derived terrestrial water storage anomaly (TWSA). Furthermore, correlation coefficients and contribution of each water storage component to TWSA are calculated to reveal inter-component interactions and response mechanisms to precipitation. Results show that original TWSA, SWSA, and GWSA increase markedly from March to July 2024. After detrending and deseasonalizing, SWSA and GWSA exhibit a complementary relationship (correlation coefficient: −0.20), with changes of −3.08 km^3^ and −1.12 km^3^ over the flood season, largely attributed to anthropogenic flood control operations. In contrast, SMSA and GWSA are weakly positively correlated (0.29), reflecting limited direct recharge efficiency. TWSA is strongly correlated with both SMSA (0.78) and GWSA (0.71), reflecting synergistic variation among water storage components. Consistently, GWSA contributes the most (44.52%) to TWSA fluctuations, followed by SMSA (31.80%) and SWSA (23.68%), highlighting the critical role of groundwater in the regional water cycle. These findings provide a valuable scientific basis for sustainable water resource management and regulation in Hunan Province.

## Introduction

Groundwater is one of the most crucial components of the global freshwater system, sustaining long-term stability in agriculture, industry, and ecosystems. However, under the dual pressures of climate change and anthropogenic activities (e.g., land-use change and groundwater extraction), groundwater systems are facing severe challenges^1^. In recent decades, significant groundwater depletion has been observed in major irrigated regions, such as the North China Plain^2^, northwestern India^3,4^, and the Middle East^5^. Such depletion can lead to a series of adverse consequences, including reduced river runoff^6^ and land subsidence^7,8^, thereby exacerbating drought and flood disaster risks. As precipitation is the primary contributor to terrestrial water storage (TWS)^9,10^, it fundamentally influences groundwater level dynamics. Therefore, quantifying groundwater storage dynamics is critical under changing precipitation patterns. However, accurate groundwater monitoring remains challenging due to limited observation accuracy^11^, sparse monitoring well networks^12^, and complex recharge mechanisms^13^.

The launches of the Gravity Recovery and Climate Experiment (GRACE) and its successor mission GRACE Follow-On (GRACE-FO) (GRACE/GRACE-FO) provide a novel approach to detect global TWS changes^14^. GRACE/GRACE-FO missions measure Earth’s time-variable gravity field to invert TWS anomalies (TWSA), enabling continuous, large-scale monitoring of water storage changes. This approach effectively overcomes the constraints of traditional in-situ observations. Over the past two decades, substantial efforts have been made to improve GRACE data processing^15–17^. TWSA has been widely applied in regional hydrological studies, including the monitoring of extreme hydrological events^18–20^ and glacier mass changes^21,22^. Since TWS integrates the vertical variation of all land-water components^23^ and groundwater constitutes a substantial part, groundwater storage (GWS) can be estimated indirectly by subtracting soil moisture storage (SMS), surface water storage (SWS), and snow water equivalent (SWE) from TWS^24^. This method has been successfully applied in numerous global and regional studies^25^. For instance, Rodell, et al.^26^ quantified the trend of global TWS changes based on GRACE data and identified groundwater depletion as a key driving mechanism. In addition, regional validations have confirmed its robustness in China, such as the Songhua River Basin^27^, the West Liaohe River Basin^28^, and the southwest karst region^29,30^.

Hunan Province ranks sixth in total water resources among Chinese provinces, yet it faces severe challenges due to pronounced seasonal and spatial disparities^31^. In 2024, the province receives 1,642.10 mm of precipitation, 13.0% higher than its long-term average, resulting in total water resources of 208.75 km³, including 45.14 km³ of groundwater^32,33^. The 2024 flood season (April-September) is marked by extreme hydroclimatic conditions. From late March to July, a sequence of intense rainfall events occurs, driven by interactions between cold and warm air masses and the northward shift of subtropical high pressure^34^. A particularly severe event in mid-June lasts for 17 days, characterized by extreme intensity (the highest average precipitation for the period since 1961), widespread coverage (affecting 14 cities), and large cumulative rainfall (e.g., 505.5 mm in the Dongting Lake area, 317% above the annual average)^35^. These heavy rains cause water levels at the Chenglingji hydrological station to reach 34.29 m, exceeding the 33 m warning level and triggering levee breaches^36^. Subsequently, from August onward, Hunan Province transitions into a drought that persists until October^37^. In this context, groundwater systems may experience significant fluctuations. Previous studies indicate that groundwater in humid regions displays strong responses to water storage variability under extreme climate conditions^38^. Given groundwater’s essential role in Hunan Province and the unprecedented nature of the 2024 extreme events, understanding its response mechanism is urgently needed.

Numerous studies have been conducted on water resource changes in Hunan Province. Huang, et al.^39^ estimated the absolute groundwater storage in the hilly areas of Hunan Province from 2003 to 2022 using GRACE data and river baseflow data. Yang, et al.^40^ revealed a seasonal reverse exchange between Dongting Lake and groundwater based on the water balance method and analyzed the disturbances caused by human activities. However, a comprehensive assessment of groundwater dynamics during the flood period, specifically using GRACE-FO data and accounting for the region’s abundant surface water, is currently lacking. This study fills this gap by estimating GWS anomalies (GWSA) for the 2024 flood season. Some studies have already incorporated surface water components such as lakes, reservoirs, and wetlands into GWSA calculations^30,41,42^. To account for the abundant surface water in Hunan Province, characterized by its dense river network, numerous lakes and reservoirs, we explicitly incorporate SWS into the GWS calculation in this study. This SWS component comprises lake water storage (LWS) from Dongting Lake and reservoir storage (RESS) from 28 large reservoirs. Specifically, we estimate GWSA by combining GRACE-FO-derived TWSA with SMS anomalies (SMSA) and SWS anomalies (SWSA). Furthermore, we quantify the contribution of each storage component to TWSA, aiming to reveal the response mechanisms and regulatory capacity of the groundwater system under extreme climate conditions. This study enhances the understanding of groundwater dynamics in humid and flood-prone regions, and provides a scientific reference for groundwater resource management and disaster mitigation in Hunan Province.

## Study region

Hunan Province is located in the middle reaches of the Yangtze River, spanning from 108°47’ E to 114°15’ E and 24°38’ N to 30°08’ N, with a land area of approximately 211,800 km². The terrain descends from higher elevations in the south to lower lands in the north. This topography favors the development of extensive river networks and numerous lakes in northern Hunan Province. Dongting Lake, one of the most important flood-regulating lakes in the middle Yangtze River Basin, serves as the hydrological center of the province, receiving inflow from four major rivers: the Xiangjiang, Zishui, Yuanjiang, and Lishui Rivers^33^.

Although the region receives abundant annual precipitation, its spatial and temporal distribution is highly uneven due to complex terrain and atmospheric circulation, leading to frequent floods and droughts. In 2024, precipitation during the flood season (April-September) accounts for 74.4% of the annual total, with 63.0% concentrating in the period from April to July^33^. Typically, high temperatures and substantial evaporation from July to September often induce drought conditions following the flood period^31^, creating a distinct hydrological regime.

**Fig. 1:**
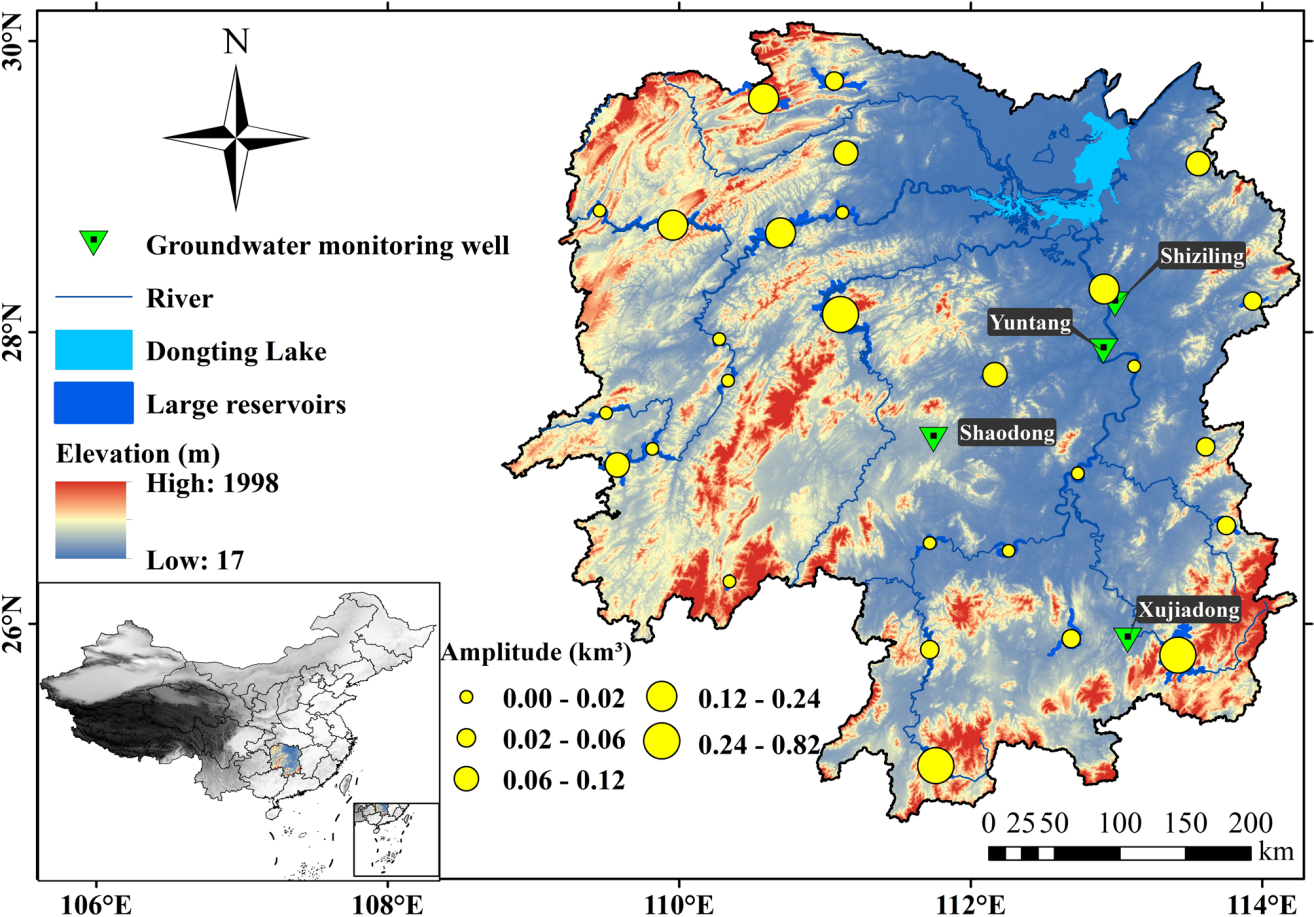
Map of the study area. The locations of four groundwater monitoring wells are indicated by green inverted triangles with a black dot. Dongting Lake and the 28 large reservoirs (individual storage capacity more than 10^− 1^ km^3^) are highlighted in blue. Note that the size of each yellow circle represents the amplitude of annual storage variation for each reservoir in 2024. The boundaries of China and Hunan Province are obtained from the National Platform for Common GeoSpatial Information Services (https://cloudcenter.tianditu.gov.cn/administrativeDivision/). This map is generated using ArcGIS Pro (https://www.esri.com/zh-cn/arcgis/products/arcmap).

## Data and method

### Data

The information of all datasets used in this study has been summarized in Table 1.

**Table 1:** The information of spatiotemporal resolution, time span, coverage and access source of datasets used in this study.

Dataset	Temporal	Spatial	Time span	Coverage	Data source
JPLM RL06.3	Monthly	0.5°	2002–2024	Global	https://podaac.jpl.nasa.gov/dataset/TELLUS_GRAC-GRFO_MASCON_CRI_GRID_RL06.3_V4
CSRM RL06.3	Monthly	0.25°	2002–2024	Global	https://www2.csr.utexas.edu/grace/RL06_mascons.html
GSFC RL06v2.0	Monthly	0.5°	2002–2024	Global	https://earth.gsfc.nasa.gov/geo/data/grace-mascons
CLDAS-V2.0	Daily	0.0625°	2017–2024	Asia	https://data.cma.cn/data/cdcdetail/dataCode/NAFP_CLDAS2.0_NRT.html
Reservoir storage capacity	Daily	-	2019–2024	-	http://58.20.42.94:9090/#/ (retrieve under “reservoir” category)
Dongting Lake water level	Daily	-	2019–2024	-	http://58.20.42.94:9090/#/ (retrieve under “river” category)
Groundwater level	Monthly	-	2020–2024	-	http://xxzx.mwr.gov.cn/xxgk/gbjb/dxsdtyb/ (latest 21-month public reports; historical data available from authors)

#### GRACE-FO data

The TWSA data used in this study is derived from the JPL RL06.3 v4 Mascon (JPLM) solutions, with the 0.5°×0.5° grid^43^. This product is applied with Coastal Resolution Improvement filter to reduce signal leakage errors. Moreover, the data are estimated by solving for monthly gravity field variations on geolocated spherical cap mass concentration functions and incorporating realistic geophysical information, thus no additional empirical destriping filter is required^44^. Additionally, CSR GRACE/GRACE-FO RL06.3 Mascon Solutions (CSRM) and GSFC Mascons are also used in this study for comparison with the JPLM-derived results, aiming to evaluate the discrepancies between the three products. The CSRM data, which have a spatial resolution of 0.25°, employ improved accelerometer processing in the RL06 release to further reduce leakage^45^. The GSFC mascon data, with a spatial resolution of 0.5°, are generated directly from Level-1B observations using a least-squares inversion for a global set of 1-arc-degree equal area mascons, incorporating a diagonal regularization matrix to reduce signal leakage^46,47^. All anomalies are relative to the 2004–2009 mean baseline.

#### Hydrological data

The China Meteorological Administration Land Data Assimilation System (CLDAS) provides hourly gridded CLDAS-V2.0 dataset, including precipitation, temperature, and soil moisture, at a spatial resolution of 0.0625° across the Asian region. After the evaluation of more than 2400 national-level automatic station observation data and the observation data of regional soil relative humidity automatic stations in China, it is confirmed that the quality of the data in China is better and the spatiotemporal resolution is higher than that of other similar products^48,49^. In this study, SMS is calculated as the sum of soil moisture content in four vertical layers: 0–10 cm, 10–40 cm, 40–100 cm, and 100–200 cm. SMSA is then derived relative to the 2019–2024 baseline.

The reservoir and lake data for Hunan Province are obtained from the Hunan Hydrological Public Service Map (http://58.20.42.94:9090/#/) produced by the Hunan Provincial Water Resources Survey Center. This platform provides real-time hydrological monitoring data for the province, including flood warnings, meteorological information, rainfall information, river and reservoir water regime, and other key hydrological information. Reservoir information is provided in the form of data recording time, water level, inflow and outflow rates, and storage capacity (unit: million m³). The platform also displays the hydrological processes of each reservoir, allowing for retrospective analysis of dynamic changes in reservoir water volume. Lake data is provided in the form of water levels (unit: m) and includes information such as flow rate and warning levels. Therefore, the LWS needs to be estimated based on lake water level data. These datasets, originally provided at daily or sub-daily intervals, are averaged to monthly values. For this study, we extract the storage capacity data of 28 large reservoirs, each with storage capacity of more than 0.1 km^3^, and the water level data from Chenglingji hydrological station at Dongting Lake from 2019 to 2024.

The 28 large reservoirs selected in this study represent the largest reservoirs in Hunan province, accounting for 90.16% of the total capacity of all large reservoirs in the province^50^. Furthermore, we compare the calculated year-end RESS data with the year-end water storage for large reservoirs published in the Hunan Provincial Water Resources Bulletin. The result reveals that the calculated reservoir water storage account for 111.15% and 146.04% of the total water storage in the bulletin respectively in 2019 and 2022, while the proportions for the other four years ranges from 94.04% to 96.56%^33^. It is important to note that the year-end water storage data in the Water Resources Bulletin uses the total water storage of all reservoirs at 8:00 AM on January 1st of the following year, while the RESS is the sum of the average water storage of 28 reservoirs during December each year in this study, resulting in some discrepancies in the recording time. Overall, the total capacity of these selected reservoirs effectively represents the total capacity of large reservoirs in the province.

Groundwater level data (unit: m) are obtained from the Monthly Groundwater Dynamics Report, which is regularly published by the water resources authority^51^. Following the completion of the national groundwater monitoring project in January 2020, groundwater monitoring capabilities have been significantly improved, including an increase in the number of monitoring stations, the spatial coverage, and the monitoring elements. Consequently, the report is revised starting in July 2020 and provides monthly averages of groundwater level and depth. Therefore, the groundwater level data used in this study cover the period from July 2020 to December 2024. The data are derived from four wells in Hunan Province: Shaodong, Xujiadong, Shiziling, and Yuntang. The locations of these monitoring stations are shown in the Fig. 1. These in-situ measurements are used to validate the GRACE-FO-derived GWSA, though their limited spatial coverage may affect regional representation.

### Method

#### Lake water storage

He, et al.^52^ used the multi-temporal remote sensing data from 1994 to 2019, combined with a variety of in-situ data, to estimate changes in the water surface area and lake capacity of Dongting Lake. Based on these results, they proposed the following empirical relationship for calculating the lake capacity of Dongting Lake by using the lake water level of Chenglingji hydrological station:

1 $${\mathrm{LWS}}= - 1.5265 \times {10^{ - 3}} \times {{\mathrm{H}}^4}+171.083 \times {10^{ - 3}} \times {{\mathrm{H}}^3} - 6.463 \times {{\mathrm{H}}^2}+106.36 \times {\mathrm{H}} - 660.28$$

where LWS refers to the lake water storage of Dongting Lake (unit: 10^− 1^ km^3^), and H denotes the lake water level at the Chenglingji hydrological station (unit: m).

#### GWSA estimates

TWSA consists of SMSA, SWSA, GWSA and SWE anomaly. SWE anomaly is neglected due to the negligible snow cover in Hunan Province. GWSA is isolated from the GRACE-FO TWSA by removing the contributions of soil moisture and surface water^53^. The calculation is expressed as:

2 $${\mathrm{GWSA=TWSA-SMSA-SWSA}}$$

where SWSA is computed as the sum of the LWS anomaly (LWSA) from Dongting Lake and RESS anomaly (RESSA) from the 28 large reservoirs. All anomalies are calculated relative to the long-term mean of each storage component over the period 2019–2024.

#### Contribution of water storage components

In order to compare the relative contributions of different water storage components to total TWS variability, the component contribution ratio (CCR) is employed, following the approach used by Kim, et al.^54^ and Asoka and Mishra^55^.

3 $${\mathrm{CCR}}=\frac{{{\mathrm{MA}}{{\mathrm{D}}_{\mathrm{S}}}}}{{{\mathrm{TV}}}} \times 100$$

4 $${\mathrm{MA}}{{\mathrm{D}}_{\mathrm{s}}}{\mathrm{=}}\frac{{\mathrm{1}}}{{\mathrm{N}}}\sum\limits_{{\mathrm{t}}}^{{\mathrm{N}}} {\left| {{{\mathrm{S}}_{\mathrm{t}}}{{-\bar {S}}}} \right|}$$

5 $${\mathrm{TV=}}\sum\limits_{{{\mathrm{i=S}}}}^{{{\mathrm{components}}}} {{\mathrm{MA}}{{\mathrm{D}}_{\mathrm{i}}}}$$

where MAD_S_ is the mean absolute deviation (MAD) of each component, and TV is the total variability calculated as the sum of MAD_S_ of all components. N is the total number of months.

## Results

### Spatiotemporal variations of water storage components

**Fig. 2:**
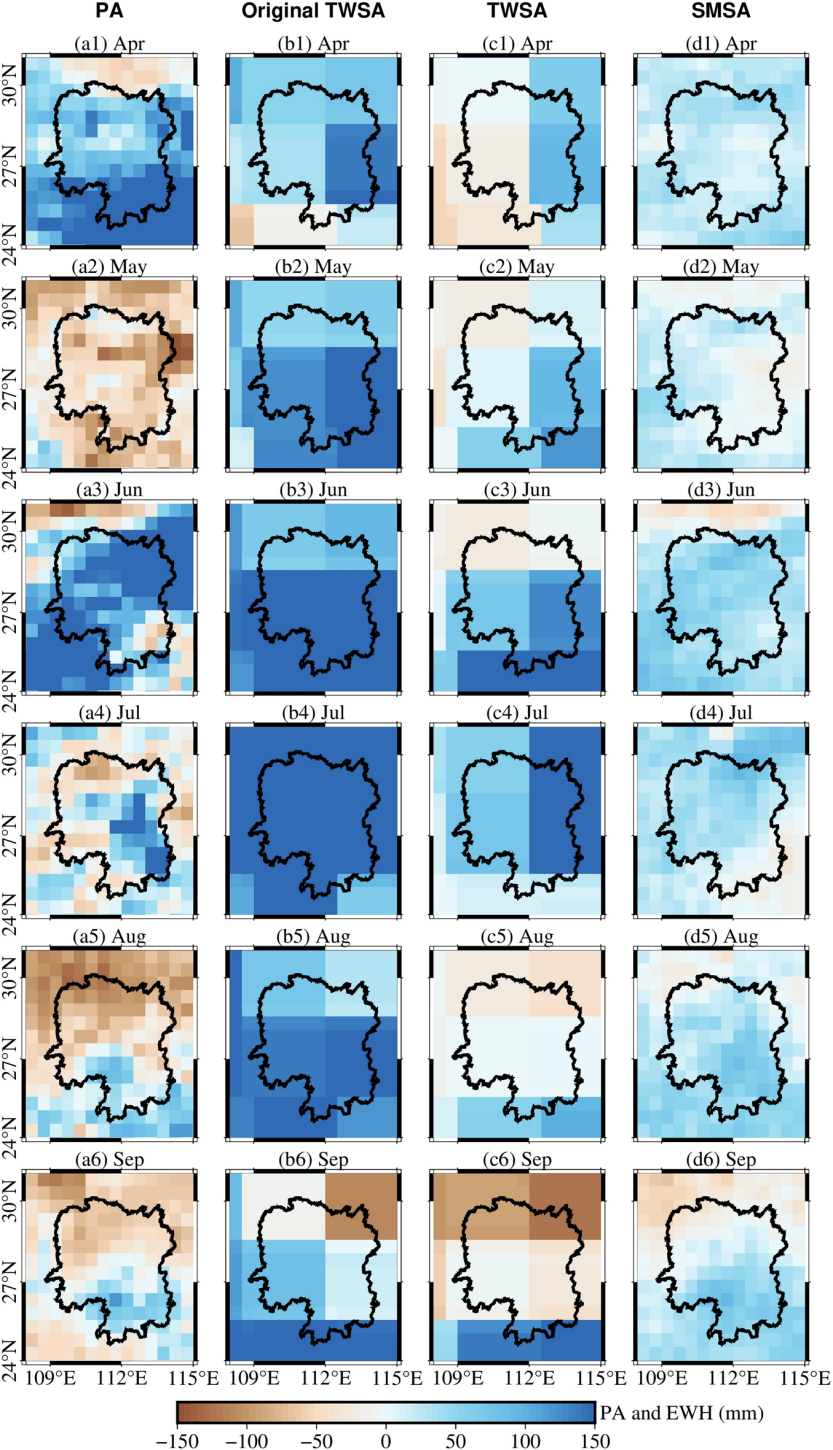
Spatial distribution of PA (a1-a6), original TWSA (b1-b6), detrended and deseasonalized TWSA (c1-c6) and SMSA (d1-d6) from April to September 2024. This map is produced using Generic Mapping Tools (GMT) 6.4.0 (https://www.generic-mapping-tools.org/).

To comprehensively assess the spatiotemporal dynamics of various water storage components during the 2024 flood season, we first present the distribution of precipitation anomalies (PA) (Fig. 2a1-a6), original JPLM TWSA (hereafter original TWSA) (Fig. 2b1-b6), detrended and deseasonalized JPLM TWSA (Fig. 2c1-c6), and SMSA (Fig. 2d1-d6) (hereafter TWSA and SMSA respectively) in Hunan Province. PA is calculated as the deviation of each month’s value from the 2019–2024 monthly climatology. Unlike the spatially continuous TWSA and SMSA grids, SWSA consists of multiple water storage changes from discrete water bodies (Dongting Lake and 28 reservoirs). Consequently, GWSA, which is derived from these components, is also analyzed as a regional time series. Therefore, spatial distribution maps for SWSA and GWSA are not shown due to lack of spatial comparability. From April to August, positive original TWSA is dominant in the province, with water storage deficits emerging in the eastern and northern regions by September.

The spatiotemporal patterns reveal distinct dynamics of water storage components in response to precipitation, which are influenced by both hydrological processes and the sampling characteristics of GRACE-FO satellites. In April, widespread positive PA, driven by alternating cold and warm air masses, leads to a marked increase in TWSA in eastern Hunan Province, while the west region experiences water storage deficits (Fig. 2a1, c1). This suggests that TWSA exhibits a delayed hydrological response. In May, precipitation is generally below historical averages, especially in eastern and southern Hunan Province, resulting in negative SMSA (Fig. 2a2, d2). However, TWSA turns positive in western Hunan, which suggests that the positive TWSA signal is released from the prior month’s heavy precipitation.

In June, heavy rainfall in western Hunan Province brings 591 mm of cumulative precipitation, causing water levels to exceed the warning threshold at multiple stations in the Xiangjiang River Basin^56^. It results in strongly positive PA and a concurrent rise in SMSA (Fig. 2a3, d3). This can be attributed to the unsaturated soil moisture and time required for water to recharge groundwater. Nevertheless, TWSA in the north remains negative in June (Fig. 2c3), while it shifts to positive in July (Fig. 2c4). We speculate that the limitation of the GRACE-FO satellite’s revisit cycle prevents it from fully capturing the changes in water storage caused by the heavy rainfall in June, resulting in this lag^57^. A late-July typhoon causes widespread rainfall in southeastern Hunan, driving positive SMSA in August (Fig. 2a4, d5)^58^. From August onward, PA becomes negative with TWSA and SMSA deficits in northern Hunan Province, indicating an emerging drought risk. These findings underscore the complexity of hydrological responses and highlight the significant impact of climate change on the spatiotemporal distribution of water storage in Hunan Province.

**Fig. 3:**
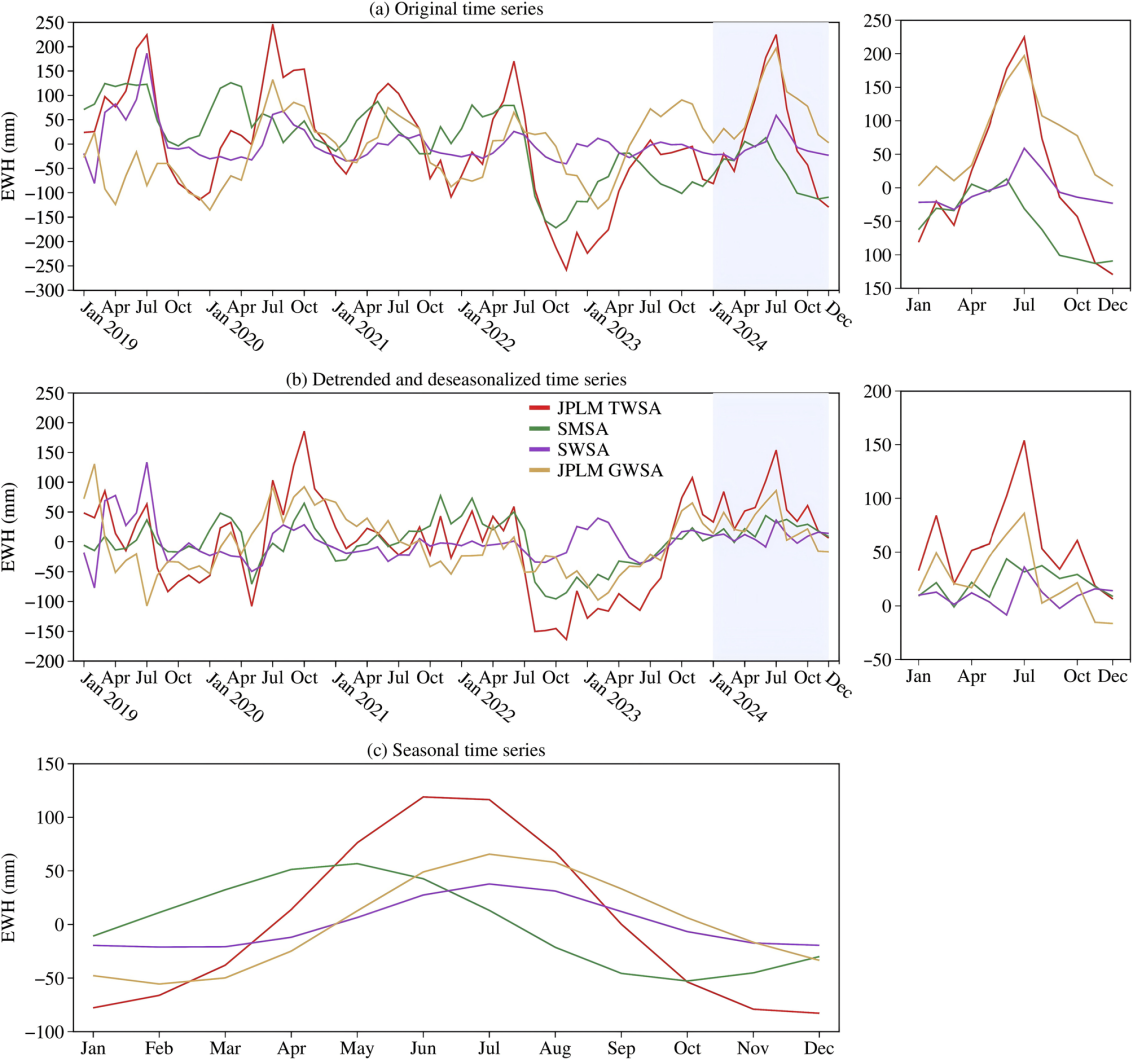
Time series of JPLM TWSA, SMSA, SWSA and GWSA from 2019 to 2024. The light purple shadow represents values in 2024, with an enlarged picture on the right. (**a**) Original time series; (**b**) Detrended and deseasonalized time series; (**c**) Seasonal time series.

This study further analyzes the temporal evolution of all water storage components from January 2019 to December 2024, as shown in Fig. 3. The original time series (Fig. 3a) exhibits pronounced seasonal characteristics for each component: TWSA and SMSA reach their annual maximum in June and May, respectively, while SWSA and GWSA both typically peak in July (Fig. 3c). In 2024, the original TWSA, SWSA and GWSA increase markedly from March to July, reaching their highest values in July. Thereafter, all components begin to decrease.

**Fig. 4:**
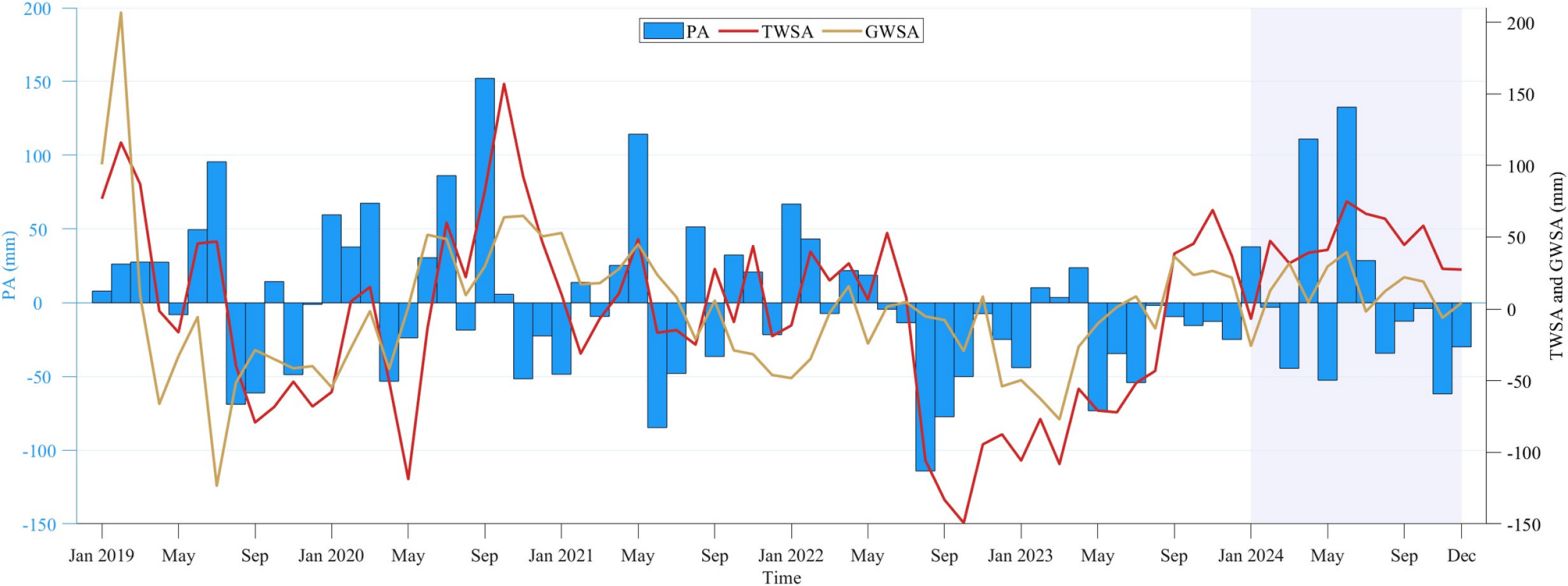
Time series of PA, detrended and deseasonalized TWSA and GWSA from 2019 to 2024 in Hunan Province.

After removing the trend and seasonal terms (Fig. 3b), TWSA and GWSA demonstrate similar variation patterns, respectively increasing from 20.86 mm to 20.81 mm in March to peaks of 153.85 mm and 85.91 mm in July. In contrast, SWSA exhibits an inverse trend to GWSA from March to June, followed by a sharp surge to a maximum (36.20 mm) in July, representing an increase of 9.45 km³. In August, TWSA, SWSA and GWSA fall by 21.29 km^3^, 4.93 km^3^ and 17.64 km^3^ (Table 2). Additionally, net changes over the entire flood season are −3.67 km^3^ for TWSA, 3.66 km^3^ for SMSA, −3.08 km^3^ for SWSA and −1.12 km³, with the dramatic monthly changes occurring in July and August (Table 2). Notably, changes in these water storage components correlate with changes in PA (Fig. 4). PA reaches 111.00 mm in April, driving high values across TWSA, SMSA, and SWSA. The maximum PA of 132.61 mm in June precedes the peak values of TWSA, SWSA, and GWSA observed in July.

**Table 2:** The water storage changes of detrended and deseasonalized TWSA, SMSA, SWSA and GWSA between two adjacent months during the flood season. ∆S is net changes over the entire flood season. Each storage component is expressed as equivalent water high (EWH) change (mm) and its corresponding water volume (V) change (km^3^).

Period	∆TWS		∆SMS		∆SWS		∆GWS	
	EWH	V	EWH	V	EWH	V	EWH	V
May−April	5.89	1.25	−13.66	−2.89	−8.45	−1.79	28.96	6.14
June − May	44.62	9.45	35.56	7.53	−12.08	−2.56	20.66	4.38
July−June	51.75	10.96	−12.10	−2.56	44.60	9.45	19.31	4.09
August−July	−100.51	−21.29	5.75	1.22	−23.28	−4.93	−83.29	−17.64
September−August	−19.09	−4.04	−11.90	−2.52	−15.33	−3.25	9.06	1.92
∆S	−17.34	−3.67	3.66	0.77	−14.54	−3.08	−5.30	−1.12

**Fig. 5:**
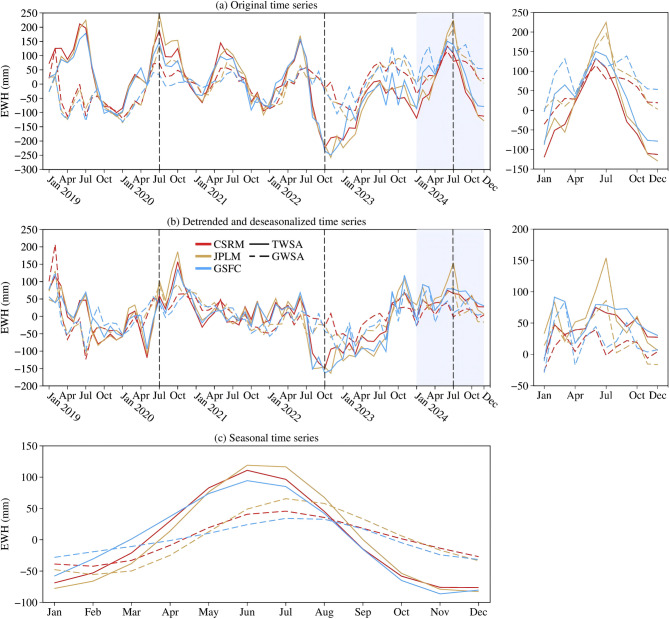
Comparison of TWSA and GWSA derived from CSRM, JPLM and GSFC from 2019 to 2024. The black dashed lines mark the time points corresponding to some extreme events.

To evaluate the impact of different GRACE solutions, GWSA is also estimated using the CSRM and GSFC products, as shown in Fig. 5. In the original time series (Fig. 5a), CSRM, JPLM, and GSFC generally show good agreement and consistent change trends. However, discrepancies are evident during periods of extreme values (e.g., July 2020, October 2022, and July 2024), where JPLM captures more pronounced extreme TWSA. Compared to CSRM and GSFC, the JPLM solution displays stronger short-term fluctuations, suggesting it may be more sensitive to extreme hydrological signals. Notably, CSRM and GSFC reach their maxima in June, one month earlier than JPLM. Although the three products display a consistent overall trend throughout the time series, JPLM more effectively highlights water storage changes associated with extreme events.

### Intercomponent Relationships and Contributions to TWSA

**Fig. 6:**
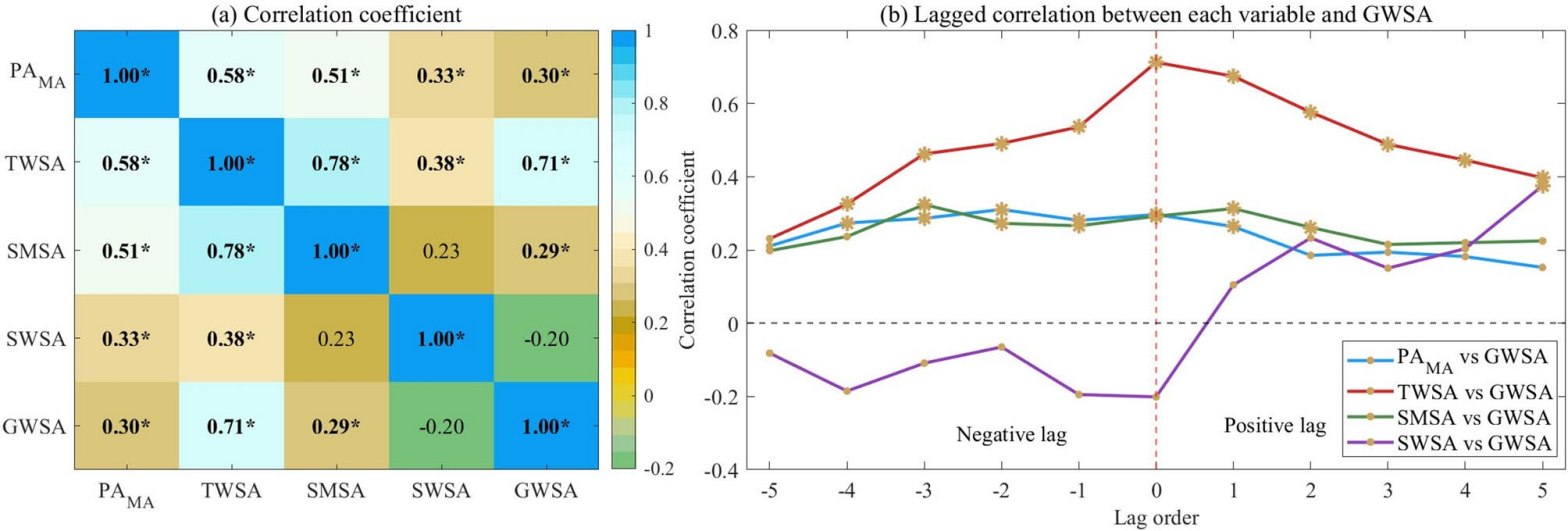
Correlation matrix among PA_MA_, TWSA, SMSA, SWSA, and GWSA, and lagged correlation between each variable and GWSA. The “*” denotes that the correlation coefficient passes the significance test (*p* < 0.05). Note: A positive lag order (i.e. i vs. j) indicates that the future (after lag order months) value of variable i is correlated with the current value of variable j, which implies j leads i. Conversely, a negative order indicates that i leads j.

The spatiotemporal variations of PA and various water storage components reveal significant differences in the responses of different components to precipitation, such as SWSA and GWSA in June. This presents the complex transformation mechanisms among different components. To quantitatively analyze the interactions, this study calculates the correlation coefficient matrix among PA, TWSA, SMSA, SWSA, and GWSA (Fig. 6a). Here, a three-month moving average of PA (PA_MA_) is used to highlight the relationship between seasonal precipitation and water storage components^59–61^. Lagged correlations between each variable and GWSA are also computed to identify potential lead-lag relationships (Fig. 6b).

Correlation analysis shows that TWSA exhibits strong correlations with both SMSA (0.78) and GWSA (0.71), its primary components, with 95% confidence intervals (CI) being 0.67–0.86 and 0.58–0.81. This indicates that these two components jointly dominate the temporal variability of TWSA. With changing lag order (Fig. 6b), the correlation between TWSA and GWSA gradually declines, signifying that no significant detectable lag effect exists in the interrelationship between these two components. Beyond this structural relationship, we focus on the connections between GWSA and independent hydrological variables to elucidate physical drivers and processes. Precipitation, as the main input source of water storage, affects groundwater through soil infiltration or surface infiltration. The correlation between PA_MA_ and GWSA is weakly positive (0.30). As lag order changes, the correlation remains relatively low. This indicates limited efficiency of direct rainfall recharge to groundwater, which is affected by many factors, such as topography, vegetation cover, and anthropogenic activities, with substantial partitioning into soil moisture, evaporation, and runoff^62–65^. Similarly, GWSA has weak positive correlation with SMSA, with a correlation coefficient of 0.29. This may result from soil moisture evapotranspiration or the geological features that weaken vertical percolation. SWSA and GWSA show a weakly negative correlation (−0.20) with a 95% CI ranging from −0.41 to 0.03, suggesting a recharge relationship between surface water and groundwater. This low correlation is likely driven by surface water management (e.g., reservoir operation) and the retention of floodwater in surface basins rather than immediate infiltration. As lag order increases, the correlation coefficient becomes positive, reaching 0.23 at a two-month lag and 0.38 at a five-month lag, suggesting that SWS changes tend to lag GWS changes by several months. This implies that surplus surface water may gradually recharge groundwater over subsequent months.

**Fig. 7:**
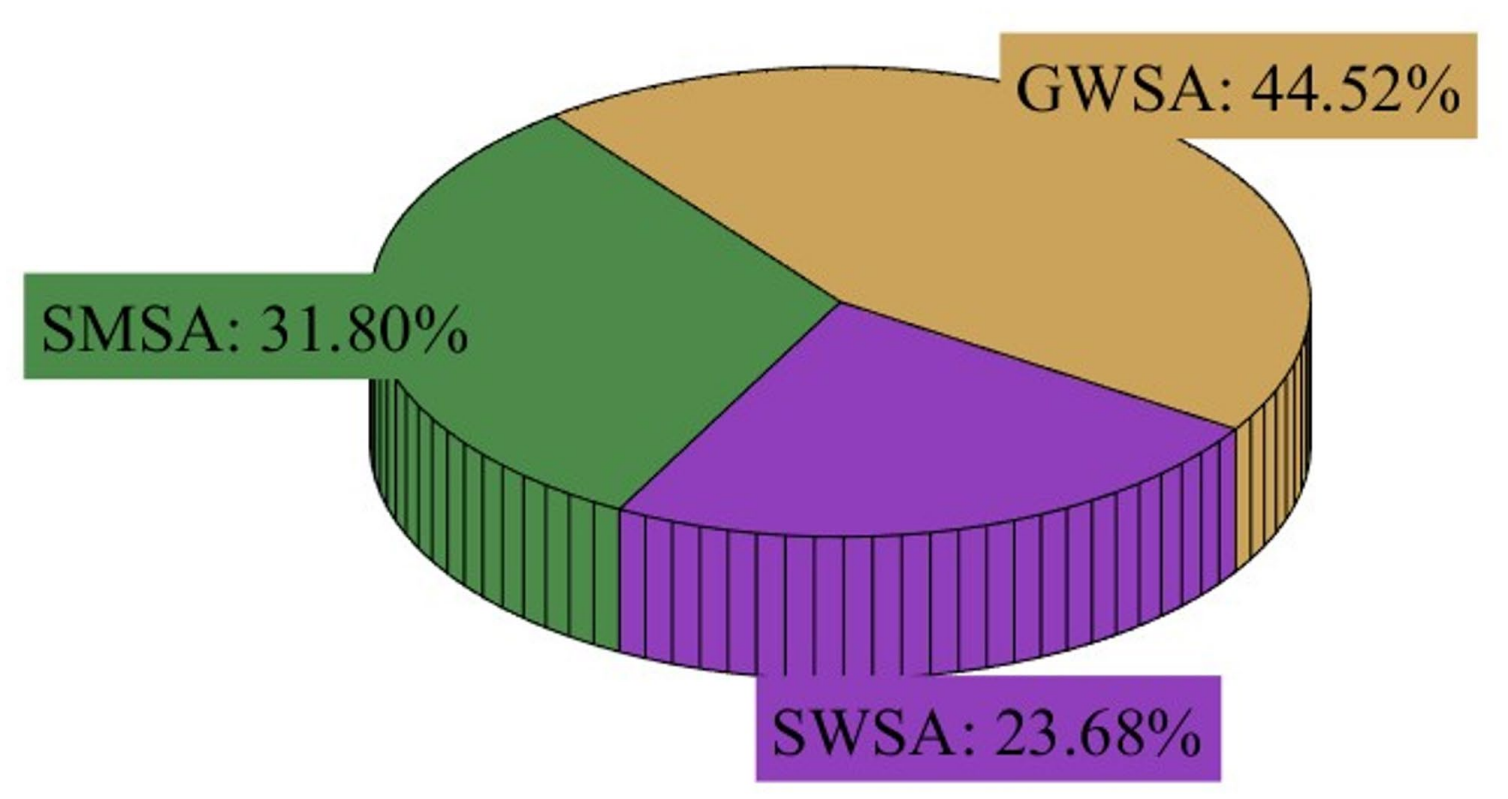
CCR of SMSA, SWSA, and GWSA to TWSA from 2019 to 2024.

Additionally, we quantify the contribution of each water storage component to TWSA using CCR. The result shows that GWSA contributes the most (44.52%), followed by SMSA (31.80%), while SWSA contributes relatively small proportion (23.68%) to TWSA fluctuations in Hunan Province (Fig. 7). This finding underscores that groundwater acts as the principal regulator of regional water storage.

### Validation of GWSA using in-situ groundwater levels

**Fig. 8:**
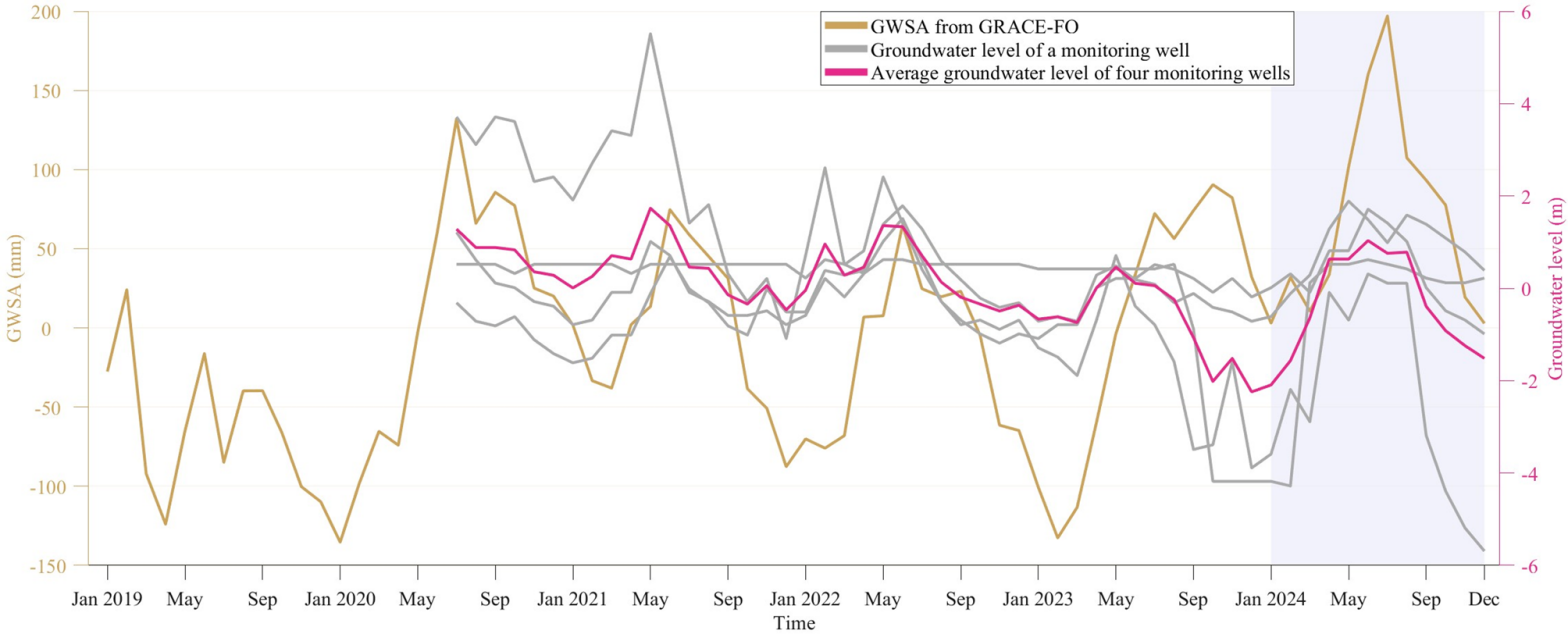
Comparison between original GWSA from GRACE-FO and in-situ groundwater level observations. The GWSA from GRACE-FO ranges from January 2019 to December 2024, while the groundwater levels from four monitoring wells (gray line) and their average (red line) range from July 2020 to December 2024.

To assess the reliability of the GRACE-FO-derived GWSA, we obtain monthly in-situ groundwater level data from four observation wells in central and southern Hunan Province (Fig. 1), and calculate their monthly average values (Fig. 8). The temporal trend of GWSA is generally consistent with groundwater level variation: they show an increase from January to June 2024, followed by a decrease from July to December.

For the period from July 2020 to December 2024, the correlation coefficient between GWSA and average groundwater levels is 0.27. This relatively low correlation can be attributed to the limited number of monitoring stations (only four wells), insufficient temporal coverage of groundwater level records, and spatial mismatch between point-scale in-situ observations and gridded GRACE-FO-derived GWSA. These limitations likely prevent the in-situ data from fully capturing the regional-scale GWS changes detected by GRACE-FO.

## Discussion

As mentioned earlier, the current GWSA estimates show a moderate correlation (0.27) with in-situ groundwater levels. This correlation is constrained by two key factors. First, the limited number and uneven spatial distribution of monitoring wells cannot adequately represent regional-scale groundwater level changes. Future studies should collaborate with official agencies to obtain more comprehensive groundwater level data for robust validation of GRACE-derived GWSA estimates. Second, the GWSA calculations contain inherent uncertainties, which must be considered. The SWSA estimates only consider Dongting Lake and 28 large reservoirs. For other lakes and medium and small reservoirs, the magnitude of individual water storage changes is smaller than that for Dongting Lake and large reservoirs. However, according to statistics, annual water storage change of 575 reservoirs (including 359 medium reservoirs) in Hunan Province, reaches −0.19 km³ (approximately twice the standard water storage capacity of a large reservoir) in 2024^33^. Existing studies have demonstrated that the mass load change of a single large reservoir can cause significant bias in regional water storage inversions^66,67^. Therefore, the cumulative water storage of these unaccounted water bodies may introduce bias in GWSA estimates. Advancements in monitoring technologies for medium and small water bodies will help reduce this uncertainty^68,69^. Furthermore, the GRACE/GRACE-FO post-processing process inevitably causes signal leakage errors^70,71^, which is particularly impactful for a study area as spatially limited as Hunan Province (211,800 km²). Hydrological signals from Dongting Lake and reservoirs along the provincial border may leak outward, while external water storage changes may contaminate signals within the province, potentially distorting the estimated anomalies^14,72^. Future studies can employ leakage correction methods to minimize signal distortion and improve the accuracy of the results^73^. Feng, et al.^74^ applied a 100-km buffer zone for leakage correction outside the North China Plain. Xiong, et al.^75^ used an independent component analysis-based forward modeling method to identify and reduce leakage errors in the Middle East.

The spatiotemporal analysis reveals a complex response of water storage components to extreme precipitation, with anthropogenic regulation being a significant influencing factor. Affected by heavy rainfall during the flood season, although the original SWSA increased from April to July (Fig. 3a), the detrended and deseasonalized SWSA decreased from April to June before surging sharply in July (Fig. 3b). This pattern is mainly caused by anthropogenic flood control. Reservoirs are pre-emptively drawn down in early summer to increase flood storage capacity, reducing RESSA (https://www.hunan.gov.cn/hnszf/hnyw/bmdt/202406/t20240626_33337014.html). At the same time, sustained rainfall and upstream discharges rapidly elevate the water level of Dongting Lake, exceeding the warning level and triggering a levee breach in Dongting Lake in July^58^. In response, reservoirs along the upper and middle reaches of the Yangtze River are urgently engaged for flood interception^76^, leading to an increase in the SWSA in July. Since June 16, key large reservoirs such as Wuqiangxi, Zhexi, and Fengtan have impounded a total of 5.86 km^3^ of floodwater^77^. To alleviate flood control pressure on the Dongting Lake, the Three Gorges Reservoir and other reservoirs in the upper reaches of the Yangtze River jointly implemented flood control measures. Starting June 27, the outflow from the Three Gorges Reservoir is gradually reduced from 24,000 m³/s to 14,000 m³/s, accumulating a total of 3.9 km^3^ of floodwater^78^. Through the joint operation of the upper and middle reaches of the reservoir group, the peak flood level at Chenglingji station in Dongting Lake is lowered by 0.20 m, and the water level is controlled near the warning level, significantly alleviating flood control pressure in the Dongting Lake system^79^. These artificial manipulations to SWS indirectly impact GWS, resulting in a low correlation coefficient (−0.20) between SWSA and GWSA (Fig. 6a). This underscores the critical necessity of incorporating SWS changes when estimating groundwater dynamics in Hunan Province.

## Conclusion

This study employs GRACE-FO-derived TWSA, SMSA and SWSA to quantify GWSA variations in Hunan Province during the 2024 flood season. Based on the geographical characteristics of Hunan Province, the calculation explicitly incorporates the water storage of Dongting Lake and 28 large reservoirs, which is critical for a region with abundant surface water. Through comprehensive spatiotemporal and correlation analysis of PA, TWSA, SMSA, SWSA and GWSA dynamics, this study systematically characterizes their hydrological response patterns and quantifies their relative contributions to TWSA variability using the CCR.

The 2024 flood season features exceptionally high rainfall in Hunan Province, particularly in April and June. While the original TWSA, SWSA and GWSA reach their maximum in July, the detrended and deseasonalized series reveal a more complex situation: SWSA and GWSA increase by 9.45 km^3^ and 4.09 km^3^ in July, whereas SMSA decreases by 2.56 km^3^. The interactions among water storage components are complex. SMSA exhibits a weak positive correlation with GWSA (correlation coefficient of 0.29), reflecting the limited efficiency in direct soil moisture recharge to groundwater. Conversely, SWSA and GWSA show a negative correlation (−0.20). This indicates that anthropogenic regulation, such as reservoir flood control, indirectly alters the relationship between surface water and groundwater. Furthermore, groundwater is confirmed as the dominant component, contributing 44.52% to total TWSA variability, followed by SMSA (31.80%) and SWSA (23.68%).

These findings enhance the understanding of how water storage components respond to extreme climate events under strong human regulation in humid regions. They provide effective decision-making support for water resources management in Hunan Province. Despite accounting for surface water such as large reservoirs and Dongting Lake in GWSA estimation, this study has certain limitations, including omission of smaller reservoirs in the SWSA estimate and potential signal leakage errors in GRACE-FO data over this spatially limited area, which may introduce biases into the results. Future research should aim to integrate more comprehensive surface water data and apply advanced correction methods (e.g., error correction models) to GRACE-FO data to further improve the accuracy of groundwater storage estimates.

## Data Availability

The datasets used during the current study are publicly available via the following official links. The JPLM RL06.3 datasets can be downloaded from https://podaac.jpl.nasa.gov/dataset/TELLUS_GRAC-GRFO_MASCON_CRI_GRID_RL06.3_V4. The CSRM RL06.3 datasets can be downloaded from https://www2.csr.utexas.edu/grace/RL06_mascons.html. CLDAS-V2.0 data can be downloaded from https://data.cma.cn/data/cdcdetail/dataCode/NAFP_CLDAS2.0_NRT.html. The reservoir storage and lake water level data are available at http://58.20.42.94:9090/#/. The groundwater level data can be obtained from the Monthly Groundwater Dynamics Report (http://xxzx.mwr.gov.cn/xxgk/gbjb/dxsdtyb/).The datasets generated during the current study are available from the corresponding author on reasonable request.
